# Surgery

**Published:** 1825-04-01

**Authors:** 


### SURGERY.

1. *Supposed Fracture of the Basis Cranii.** H. Gilchrist, aged 50, a labourer, was suddenly thrown from a brewer's cart, on the evening of the 8th May, 1821, and fell insensible to the ground. On being raised, blood was seen to issue from his ears to a considerable extent. Two hours after the accident, he was seen by Mr. Macfarlan, and the following symptoms noted.

" He is somewhat comatose, but, when roused and spoken to, answers, though not very distinctly, and rather incoherently. Complains of pain of head, especially when touched, and chiefly behind the right ear, where there is an evident softness, and slight fullness; but no de-

* J. F. Macfarlan, Esq. Ed. Transactions, vol. I.

pression of bone, nor other injury, can be discovered in any part of the cranium. There is no external wound. The right eye seems sunk in the orbit; whilst the superior palpebra is partially paralysed: both pupils are dilated, but contract readily on the approach of light. There is still a considerable hæmorrhage from both ears, the blood flowing in a constant small stream. He also complains of pain of the side, when touched, near the cartilage of the fourth rib, but no injury can be perceived. Has had some vomiting. Pulse 48. Skin cool." 652.

He was visited again in two hours, when he was found in nearly the same state, but more easily roused. There appeared to be no loss of muscular power, as he was impatient of controul, and was kept in bed with difficulty. Twenty-five ounces of blood were taken from the arm, after which the pulse rose to 57. 9th. Passed a tolerably quiet night, though with some retching and startings—hæmorrhage from the ears continues—complains of his head—no motion in his bowels. Temporal arteriotomy to fourteen ounces—head shaved, and cold applied. 10th. Was delirious last night—pulse 50, and feeble. 11th. A bad night—headache—pulse 50—no evacuation yet—venesection to twelve ounces—five grains of calomel and ten of jalap. 12th. A bad night—answers questions more distinctly—pulse 50, and irregular—bowels open. 13th. A good night. 15th. A restless night, with low delirium—pupils dilated—pulse 64—passed urine involuntarily. 18th. A bad night, with constant delirious muttering—urine and fæces passed in bed—eighteen ounces of blood from the arm—pulse 120, and feeble—some paralysis of the face. After several days of changeable and formidable symptoms, appearances became better, and he recovered to a certain extent. His memory, judgment, and corporeal powers being still much impaired.

"The case, as I have hinted, appears to belong to that particular species of counter-fracture first described by Dr. Thomson, in the 8th volume of the Edinburgh Medical and Surgical Journal. All the leading symptoms mentioned by this acute observer, such as hæmorrhage from the ears, stupor, and diminished frequency of the pulse, were present; and when to these we add other subordinate circumstances of the case, namely, the sunk appearance of the right eye, the swelling and discoloration behind the ears, the paralysis of the face, the involuntary discharge of fæces and urine, together with the derangement of the intellectual faculties—little doubt, I imagine, can remain, but that such was the true nature of the injury. From the foregoing narrative, therefore, encouragement is given to our perseverance in affording relief, even under the cheerless prospects that this variety of accident generally presents." 657.

---

2. *Treatment of Hernia** Twenty-four cases of strangulated hernia have presented themselves to Mr. Yeatman, and all been treated with

* Mr. J. C. Yeatman. Med. and Phys. Journal, Jan. 1825.

success. Twenty one of these were reduced by the taxis and remedial measures: while three were operated on at an early period of strangulation. Fifteen of the twenty-four were cured by venesection—the application of cold—and the taxis—six cases by the above means, in addition to five grains of opium, exhibited at one dose internally. In all the cases, the gut had been strangulated from one to five hours. Each patient was immediately bled *ad deliquium*—then placed on the floor of the room, on a blanket, with pillows to raise the pelvis, and the taxis managed in the usual way. Pails of cold water, with ice, or muriate of soda and ammonia dissolved in water, were placed by the side of the patient, and poured on the hernial tumour, at the distance of two feet, from the spout of a large tea-pot, or small tea-kettle, for a space of time varying from an hour to an hour and a half—occasionally employing the taxis for a few minutes together. When this mode of treatment failed of success, five grains of opium were given in each case—the patient put to bed—and the taxis had recourse to during the profound sleep that followed. The above were the only means employed by our author—and always with success. With respect to the operation, he considers it disgraceful to a surgeon to find the contents of the hernial sac in a state approaching to gangrene—when he might have operated sooner. But, says he, all the remedies which have ever been recommended, are generally put first in execution, and many hours and days are thus suffered to elapse "in imbecile and reckless attempts to return the inflamed, enlarged, and strictured parts into the abdominal cavity."

---

3. *Iritis.** We can only touch on two of the points discussed by Dr. Robertson—namely, the etiology and treatment of the disease in question. He observes that almost all authors have been surprized that "mercury both causes and cures iritis," and so they might, he thinks, were this a fact. It appears to him that "mercury *alone never* causes the disease." It may act as a *predisposing*, but not as an *exciting* cause. Mercury renders the constitution more liable to the impressions of *cold*—this last being the most frequent cause of iritis. The following are the facts on which our author grounds his conclusion.

"1st. The greater number of patients that I have had an opportunity of seeing affected with iritis, while their systems were under the influence of mercury, were conscious of having been exposed to cold shortly before the attack.

"2nd. In those cases, other effects, considered as arising from cold alone, were present, such as an increased discharge from the nares and fauces, rawness of the throat, &c.

"3rd. Inflammatory affections of other organs are frequently caused by exposure to cold, under similar circumstances; as inflammation of

* Dr. J. A. Robertson. Ed. Journal, Jan. 1825.

the lungs, bronchiæ, and intestines, none of which have ever been considered as produced by mercury alone; and lastly,

"4thly. In the greater number of iritic cases that have been under my care, the patients neither were, nor had lately been under the influence of mercury, and nearly all of them attributed the complaint to cold. Besides, if mercury caused iritis, this disease ought to have been of more frequent occurrence some years ago, when ten times as much mercury was used as now a days: but I do not find that this was the case." 34.

*Treatment.* At the commencement of the attack, blood-letting is the first remedy to be employed—the quantity being proportioned to the effects produced on the system, and on the disease. When the system has been sufficiently lowered, we should have recourse to topical bleeding and blistering.

"The medicine, however, that is chiefly to be relied on in iritis, is mercury. And to Dr. Farre, who was the first in this country who introduced the use of mercury in iritis, we are indebted for this most important improvement in the treatment of this disease. But the use of this medicine in iritis was known to Bëer, Benedict, and other German oculists, long before its introduction into this country. In iritis, the system is to be affected with mercury as rapidly as possible; for this purpose, in severe cases, I frequently order two mercurial pills to be taken every hour combined with opium, if they affect the bowels with griping or purging. The supra-orbital region is to be well rubbed twice a day with mercurial ointment, mixed with powdered opium. The relief from pain which the patient often experiences from the latter application is quite astonishing. As soon as the system becomes affected with mercury, the patient experiences a very considerable abatement of the pain and feeling of fulness and distension of the eye. The sight becomes clearer, and the irregular and contracted state of the pupil and the effused lymph begin to disappear. The redness of the external parts diminishes, and the iris assumes its natural colour. These effects are almost invariable; indeed, I do not remember a single case in which the pain was not relieved as soon as the system was affected." 36.

When lymph has been effused, mercury is indispensible—it is the only remedy which can be of any service. It is to be aided by the application of belladonna, or hyoscyamus externally.

---

4. *Mental Imbecility from Injury of a Nerve.** A young man, 14 years of age, of plethoric habit and nervous temperament, received an injury on the top of the foot, from a stone thrown with violence by one of his play-mates. By this accident, a portion of the anterior tibial nerve, situated between the metatarsal bone of the great toe and the adjoining one, was contused. Only a slight degree of pain at first oc-

* Dr. James Anderson. New York Medical and Physical Journal, No. 4.

curred, and that upon motion. About eight or ten weeks after the accident, the pain increased, accompanied by a slight swelling of the part, and also an extension of the pain to the ancle and leg. Lotions, fomentations, and rest did no good, and the disease increased in violence, extending up along the trunk of the nerve, and affecting the adjacent muscles with spasms. The constitution now became involved in the irritation—the pulse increased in frequency and force, but the digestive organs continued in good order. The pain extended above the knee, and was almost insupportable. Large doses of assafœtida, conium, and even belladonna produced no sensible relief. The symptoms became more formidable—the spasmodic action of the extensor longus pollicis pedis drew the great toe perpendicular to the surface of the foot—and the slightest motion or touch would excite excruciating pain, lasting from three to five minutes. At the end of three months from the accident, the whole nervous system became involved in these spasmodic irritations, and the intellect began to suffer. The unfortunate patient lost the power of reasoning, and recollection—was unable to distinguish visitors, or recognize his parents—in fact, his mind became idiotic. The pain, however, now extended up along and beyond the thigh, affecting spasmodically the muscles of respiration. Though his distress was most acute, yet he gave no utterance to his feelings. "While the paroxysms were on him, he would roll his fist, and imitate the actions of a pugilist, but with much greater violence and rapidity, often striking his nearest and best friends, and all around him." It was now determined to make a division of the anterior tibial nerve, as a last resource. It was accordingly divided above the ankle, from a belief that it would more effectually cut off the connexion between the injured part and the nervous system at large. An incision was made about two inches in length, just on the outside of the tibia, and a separation made between the extensor longus pollicis pedis and the tibialis anticus, down to the artery and nerve. The latter was divided by a bistoury, and about an inch of it cut out. The extensor muscle of the great toe became immediately so relaxed, as to allow the toe to resume its natural situation by the side of the other toes. There was also an almost immediate alleviation of all those extraordinary morbid phenomena that had exhibited themselves in the unfortunate sufferer. The local affections became less distressing and in a short time, wholly disappeared. He has since enjoyed good health.

Although Dr. Anderson does not mention any thing about the return of sound intellect, we take it for granted that this was the case, otherwise he would not have stated the enjoyment of *good health*. The case affords a curious instance of the power of a local affection in deranging the health of body and mind.

---

5. *Ophthalmia.* In a report of ocular diseases from Mr. Melin, assistant surgeon to the forces, and on duty at the General Hospital, Fort Pitt, we find a novel method of treating ophthalmia, which we sincerely hope will prove efficacious in the hands of other practitioners. There is something revolting in the practice of detracting large quantities of blood from the general system for the removal of a purely local inflammation. Any mode of curing ophthalmia, therefore, by local means, is a desideratum. The disease, as it occurred at Chatham, was certainly mild, comparatively speaking, and did not present itself in the form commonly called Egyptian. From having witnessed the good effects of a solution of lunar caustic, in allaying the pain and suppressing the discharge of gonorrhœa, Mr. Melin was led to try its effects in inflammation of the conjunctiva, a membrane similar to that lining the urethra, and where stricture was not to be dreaded. The opportunity soon offered of putting his ideas into action. Four grains of the caustic were dissolved in the ounce of distilled water, a little of which was dropped into the eye twice a day. It excited pain, and a sensation of roughness, with an increased flow of tears, for about ten or twenty minutes, after which the eye felt much relieved, and in a few days the cure was effected. In this way he has treated nearly 300 cases of acute ophthalmia, some of them of a severe nature, without either local or general bleeding. "One very material advantage attending this mode of treatment is, that the inflammation is subdued without leaving any chronic disease, either in the eye-balls or lids." This is not always the case when the antiphlogistic plan is employed. Some of his professional friends have given the method in question a trial, and with similar success. It is proper to observe that, where there is deep-seated pain, and suspicion of iritic inflammation, depletion must be employed. It is to the conjunctival inflammation that the caustic treatment is applicable.—*Med. and Phys. Journ. Sept.* 1824.

In the October number of the same Journal, Mr. Melin continues his ophthalmic report.

*Iritis.* Five cases of this complaint were admitted, of which three were cured, and two invalided. In the latter, the eyes were disorganized or the retina insensible before the patients came into hospital. In these cases of iritis, as well as in others, our author immediately commenced the use of mercury, giving two grains of calomel with a quarter of a grain of opium every two hours, until the mouth became affected; "on which a visible and marked improvement took place in the disease. The aqueous humour became clear, the absorption of effused lymph commenced, and the pupil recovered its round form."

"Indeed, I know of no complaint in which the action of medicine is so clearly and beautifully seen as in this. I shall not enter into the question of the various species of iritis, whether idiopathic, syphilitic, or mercurial; but, in whatever form it appears, or whatever cause produces it, I must express my unqualified confidence in the power of mercury (when properly and judiciously administered,) to arrest its progress, and effect its cure. In general, I consider that, the more rapidly the system is placed under its influence, the better; and I have always commenced with two grains of calomel and a quarter of a grain of opium, in a pill, every two hours. But a case has lately occurred here, in a poor patient who applied for my advice, which will lead me to increase the quantity when next I meet with the complaint. The eye was painful, the sclerotic coat very vascular, the pupil irregular, the aqueous humour turbid, and a small quantity of lymph effused into the anterior chamber. I directed him to take the pills as above; and the next day he called on me, when, to my surprise, the eye was nearly perfectly recovered, and his mouth very sore. On inquiring I found that, by mistake, he had taken two pills every two hours. I ordered him to discontinue the pills, and directed a wash for the mouth; and on the following day there was no sign of the disease." 272.

A very curious case of periodical iritis is related by Mr. Melin, which is worthy of attention. The patient was of delicate habit, and had recently been discharged from the medical division for a rheumatic attack. The iritis came on 14 days after being stopped by calomel and opium—then ten days—then nine,—and afterwards, every seven days after its arrest. The periodocity of the complaint was now so unequivocal, that Mr. Melin had recourse to the bark. Still it returned on the 7th day, but was less severe, and continued to do so regularly for three weeks. The liquor arsenicalis was then administered. This medicine had the desired effect, as only two slight weekly paroxysms occurred afterwards.

*Ulcers of the Cornea.* When these were of a spreading nature, our author paid particular attention to the general health, as in most cases they depended on it. They principally occurred in week and delicate constitutions, or where the system had been much lowered by bleeding, purging, &c. In such cases, much benefit was derived, after clearing the primæ viæ, from bark and mineral acids, and gently touching the edges of the ulcers with a piece of caustic scraped to a fine point. When the ulcers were very irritable, he directed the eye to be held over the vapour of boiling water, to which were added some tincture of opium and camphor mixture. This generally afforded much relief.

At the conclusion of his ophthalmic report, Mr. Melin has the gratification to add that, out of 568 cases of disease, there was not an eye lost, nor one impaired, that was not irrecoverably so before admission into hospital. In 37 cases of operation, only one could be said to have fairly failed. This report forms a good appendix to the ophthalmic article in this number of our Journal, furnished from the works of Dr. O'Halloran and Mr. Hewson.

---

6. *Anchylosis of the Jaws.* Mr. Snell relates a curious case of this kind, which he lately met with in the country. A little girl, eight years of age, had her jaws completely locked for two years past. A tumour having formed on the left side of the lower jaw, some practitioner rather incautiously attempted to cure it by pressure, for which purpose "he bound up the head and jaw tightly with a bandage and compress." Inflammation having attacked the jaw and face, complete anchylosis of the joints of the jaw, was the ultimate consequence. Notwithstanding this state, the little girl was the picture of health, and managed to rub in very quickly a sufficient quantity of bread and butter through the interstices of the teeth, to satisfy nature and preserve health. Milk, her only drink, was readily sucked in through the same channels. By extracting some of the temporary front teeth, the little patient became so satisfied with her increased (though temporary) facility in eating, that she refused any further mechanical aid.—*Med. Repos. Feb.*

---

7. *Recto-vesical Lithotomy.* This operation was first practised in France, where it has been very coldly received. It was imported into Italy, and there it seems to take root and thrive. It has been practised at Turin, Genoa, Milan, Pisa, but in different modes. Some surgeons have abandoned it after trial—others have written against it on theoretical grounds, and without trial—while the greater number who have performed it, give it the preference to either the lateral or the high operation. There are some solid and important facts connected with this subject, which are not undeserving of attention. The following are some of them. Of 69 who have undergone the recto-vesical operation, 13 have died, or rather less than one in five. Of these 13, seven are asserted to have died of affections totally independent of the operation, 56 have been cured—but out of these 56, there are eight with fistulous openings. Of these eight, one had the lower part of the bladder (le bas-fond de la vessie) incised. Forty-eight therefore have been cured without fistula, in four of which the " bas-fond" had also been injured. The period of recovery varied from ten or twelve days to seven months. Seven were cured between the eighth and fifteen days—twenty-eight between the fifteenth and thirty days—ten between the 30th and 60th days —and eleven between the 60th day and seven months.

The principal advantages of the recto-vesical operation are considered to be—the impossibility of wounding the pudic artery—the absence of hæmorrhage during and subsequent to the operation—the facility of extracting the largest calculi—the little risk of urinous infiltrations. On the other hand, the serious inconveniences of this operation are candidly stated, and are as follows:—the wounding of the seminal vessels—and the chance of recto-vesical fistula. It is yet to be proved that the *first* accident entails impotence.* In respect to the *latter*, it is said that it will be very rare where the incision does not go beyond the limits of the prostate gland. It will be frequent, but not *inevitable* in those cases where the incision reaches the lower part (bas-fond) of the bladder.

---

* We know an instance where, after a successful lithotomy in the lateral way, the seminal vessels have been obliterated, and all communication with the urethra cut off. The inconveniences are rather distressing, though the gentleman is upwards of 70 years of age. It is probable that inflammation about the verumontanum was the cause of this occlusion of the seminal vessels —*Rev.*

In the lateral operation, there is little danger of either fistula or wound of the seminal vessels; but then there are other dangers and inconveniences, viz. 1mo Injury of the rectum—an accident by no means extremely rare, even in the hands of the most expert operators. 2ndo. Hæmorrhage, examples of which are, unfortunately, but too frequent—and generally mortal where the pudic artery is divided. 3tio. The impossibility of extracting a very large stone by the lateral operation. 4to. The greater mortality by the lateral than by the recto-vesical operation. This parallel Mr Breschet has drawn from the memoirs of Vacca and others in Italy, and he comes to the conclusion that—the recto-vesical operation is, or appears to be, preferable to the lateral—and that it ought to be more frequently practised than it now is in France.—*Archives Generales, Juillet,* 1824.

P. S. As many of the readers of this series may not be acquainted with the method of performing the recto-vesical operation we shall give a very short description of it in this place, in the words of a transatlantic writer, Dr. Muter, of New York, formerly of Wisbeach in Cambridgeshire, who has published a little work on the subject of Lithotomy, which does much credit to his judgment as an ingenious surgeon. There are two modes of performing this operation, one of which may be called the high, and the other the low recto-vesical incision. The former, we believe is now seldom practised. It consists in an incision made through the rectum and bladder, above the sphincter of the one, and prostate of the other, into the slit or groove of the staff, in the place where the bladder is punctured for suppression of urine—cutting a little to one side, to avoid wounding a small artery which runs along the middle of the anterior surface of the rectum.

" There is another situation, says Dr. Muter, towards the verge of the anus and perineum, where a somewhat different operation may be performed, and a very large stone extracted. If I have maintained that a stone of small size only, ought to be extracted by the last, by this, I now assert that *only a large one* should be extracted."

" We are told by Professor Vacca, that the fæces may be excluded from entering the bladder, by this mode of forming a valve of the rectum, opposite the wound in the prostate and neck of the bladder. The incision is to be made anterior to the prostate, and carried through the rectum, urethra, and sphincter of the anus, an inch into the perineum, cutting along the slit in the staff. The surgeon then runs the knife along the groove in the staff, and dividing the prostate and cellular membrane, leaves the rectum uncut. This valve of the rectum extends about an inch lower than the opening into the bladder." Dr. Muter observes that, it must be exceedingly difficult to avoid including the rectum also in this incision, in which case there would be no valve. And, even granting that the surgeon succeeds in forming the valve, Dr. M. thinks that it would be in the way of extracting a large stone, forming a stricture of the wound, and impeding the exit of the calculus. Besides this, it would, he thinks, be very liable to be torn. The above objections of

Dr. Muter, let it be remembered, are not from actual experience of their validity, but from reasoning on the subject.

---

8. *Staphyloma cured by Tapping.** Dr. Richard Martland has related a case of this kind, of which we shall present some of the particulars. J. Snape, ætat. 38, was afflicted with severe pain in the head, while in his Majesty's service at Gibraltar, in 1812, terminating in quotidian intermittent, of two months duration. In April, 1820, Dr. M. was consulted, and found a cataract of both eyes. In October he cut up the lens of the right eye, and pushed a small portion through the pupil. The operation was repeated twice, and with complete success. On the 14th December, an oculist extracted the cataract from the *left* eye, with some difficulty, owing to a partial adhesion of the capsule to the iris, which was unfortunately lacerated, with escape of some of the vitreous humour. Prolapsus of the iris, with violent inflammation took place the first day after the operation, and continued upwards of six weeks, leaving the patient quite blind of this eye, though very active measures had been pursued to reduce the inflammation, and the unreduced portion of the iris was destroyed by lunar caustic. He again applied on the 30th April, complaining of pain darting through the left temple and forehead, with sense of throbbing as well as of pain at the bottom of the orbit of the left eye. The conjunctiva was very vascular, and a large tumour was situated on the ball of the eye, opposite the external canthus, when the eye was directed to the opposite side. The tumour resembled that of the sclerotic coat delineated in Travers's Synopsis, pl. 1. fig. 7, but was rather broader at its base, and of a deeper colour. The iris lay very near the cornea, and had become adherent to it where the incision was made. After some preparatory measures, the eye was tapped by means of a small cataract needle, and some of the vitreous humour escaped. The pain was relieved in half an hour; but recurred in the night as bad as ever. The inflammation of the eye became more extensive, and the tumour increased in size. Bleeding, purging—calomel, opium and antimony. In about three weeks another puncture was made, and a considerable quantity of viscid fluid escaped, with much relief. The inflammation now subsided, and the man resumed his trade of weaving. In a few days the tumour again filled (there were now two of them) and the headache returned. They were again tapped, and again much viscid fluid was evacuated, with instant relief. In a week all the bad symptoms recurred, and repetition of the paracentesis brought the usual good effects. We cannot follow the author through his diurnal detail of symptoms and operations; but on the 16th August, we observe that Dr. M. varied the paracentesis. He made a perpendicular incision through the coats of the eye, fully one third of an inch in length, and about two lines and a half

---

* Ed. Journal, January, 1825.

from the temporal edge of the cornea. A great quantity of vitreous humour soon escaped, causing the eye to sink a good deal in the socket, and become very flabby. In spite of this the tumours soon filled again, and it was necessary to tap them six or seven times afterwards. On the 23d February, 1824, no vestige of the tumours remained, and there is a cicatrix where the incision was made. There has been no return of the pain or inflammation.

---

9. *Knife Swallowing.** Our readers must remember the remarkable history of Cummings, the knife-eater, as related by Dr. Marcet and others. The present case was the more pitiable, as the knife was swallowed involuntarily. The unhappy victim was a juggler, 28 years of age, who, while feigning the swallowing of a knife, did actually swallow it, having, by accident, let it slip out of his fingers. The knife measured nine inches in length, having a bone handle, which went foremost down the œsophagus. Various attempts were made by the surgeons in Carlisle (where the accident happened) to extract the instrument, but all to no purpose. The knife could not be felt in the throat, nor yet in the region of the stomach. This happened in the evening of November 17, 1823. Although the man was dreadfully alarmed, he did not feel any considerable pain or inconvenience at first. He was directed to keep very quiet, and to take nothing but a little cold water that night. He had some sleep, and next day experienced some pain in his stomach. The handle of the knife soon became distinctly tangible a little above the umbilicus, when the stomach was empty; but on taking food or drink, the distention of the stomach prevented the knife being felt. His sufferings were not so great as might have been expected, but still his health declined and his strength became reduced. He was able to walk about a little in the day, and could sleep at night, on his back, but not on either side. The alvine evacuations were of a dark ferruginous colour; the pulse was little affected. His diet consisted of soup, gruel, and tea, taken in small quantities. He was frequently squeamish and sick at his stomach, sometimes experiencing a severe twisting pain in that organ.

Various projects of relief were made, but none put in execution. Sir Astley Cooper was consulted, and, we believe, he recommended to cut down on the knife and extract it. The surgeons of the Carlisle Dispensary were of the same opinion unanimously, and we think they were right. Any attempt at extraction by the œsophagus we should consider as much worse than useless. Had the patient lived long enough for such dissolution of the blade as to allow the remains to pass the pylorus, death would, in all probability, ensue, from the entanglement of the ragged remains in the intestines, as was the case with his unfortunate predecessor, Cummings.

---

* Case of W. Dempster, who swallowed a table-knife, nine inches long. By Thomas Barnes, M.D. [Ed. Philosoph. Journal.]

The patient remained at Carlisle till the 28th December, when he left that place, contrary to the advice of his medical attendants, for Hammersmith. He never reached his destination. The motion of the vehicle brought on inflammation and gangrene of the stomach, of which he died at Middlewich, in Cheshire, on the 16th January 1824. No authentic account of the dissection has been published by the surgeons who opened this patient, but we hope the deficiency will be filled up, when this public call reaches the parties concerned.

A case somewhat similar to the above is quoted by Dr. Barnes from a small latin work, published at Leyden, in 1636, by Dr. Beckher. The particulars are as follow:—A young peasant swallowed a knife, with the handle of which he was irritating the fauces to provoke vomiting. He was much frightened, but yet he was able to follow his usual avocations without much inconvenience. A meeting of the faculty having been held, it was agreed that the abdomen should be opened, an incision made into the stomach, and the knife extracted. A straight incision was made in the left hypochondrium, two fingers breadth under the false ribs, through the peritoneum. The stomach subsided and slipped from the fingers, but was at length caught hold of by means of a curved needle, and drawn out of the wound. An incision was then made into the stomach, and the knife was easily extracted. The stomach immediately collapsed. The external wound was united by sutures. The patient had a quiet night—but passed some blood in his urine. The wound went on well, and on the seventh day the patient was pronounced out of danger.

We may conclude with a remark which we formerly made on the practice, now occasionally adopted, of publishing medical and surgical cases in periodical works of *general* literature. It is, we imagine, a practice of more than questionable propriety. It is little better than an advertisement in a newspaper. The proper channel is through the medical press—and he who brings professional cases before the world at large—*may* make the vulgar stare, but certainly *will* make the sensible grieve. We hope to see the practice abolished.

---

10. *Retention of Urine.** On the 5th of August, Mr. Thompson was desired to visit J. Watson, aged 54 years, who had been suddenly seized with retention of urine the evening before. Various means were used to cause the urine to flow, medical and mechanical, but in vain. The bladder became much distended, reaching almost to the umbilicus, while the greatest agony was experienced by the patient. The distended bladder pressed so much on the rectum that an enema could not be thrown up, nor the bowels emptied. In this condition, delirium came on, with other threatening symptoms, and it was determined to puncture the bladder above the pubes. The skin was first cut with a lan-

* Mr. Thompson, of Whitehaven. Repos. December, 1824.

cet, and then a large-sized trochar pushed into the bladder obliquely. Nearly a chamber-potful of urine was drawn off. A flexible catheter was then passed through the canula, and both the instruments secured by a tape round the body. The urine flowed entirely by the tubes for six days, at the end of which a little was made in the natural way. In three weeks, the instruments were removed, the whole of the urine now passing *per vias naturales*, and that in a fuller stream than he had seen for some years.

Mr. Thompson makes some remarks on the three ways of puncturing the bladder, preferring that above the pubes to that by the rectum or through the perineum. He recommends that the trochar be either pushed at once into the bladder, or a small puncture first made with the lancet, so that the skin and integuments shall afterwards grasp the canula, and prevent the escape of water by the sides of the tube.

From what we have seen ourselves, we decidedly prefer the suprapubian operation. But we may remark that, when the retention of urine arises, which is generally the case, from a stricture in the urethra, there is no occasion to puncture the bladder at all. An opening should be made in perineo, and the urethra pierced behind the obstruction. This wound will afterwards heal, by proper care, when the urine finds its natural course.

---

11. *Paracentesis Capitis.** We have long been of opinion, that where hydrocephalus has proceeded so far as to admit the operation of tapping, it is incurable. The following case, published by Mr. Money, will not weaken this position.

J. R. Hunt, ten months old, came under Mr. Money's care on the 15th October, 1822. Even at birth his head was unusually large, and the size gradually increased—his health became deranged—and medical advice was sought. Under Dr. Webster's care the general health was restored, but the head still increasing in size, Dr. W. considered the case hopeless, and Mr. Money, by and with the advice of council, determined on paracentesis capitis. The relative weights of the head and body at this time (as ascertained without decapitation) was as follows:—body 14 pounds 10 ounces—head six pounds and a half.—The various admeasurements of the head we shall pass over. The temperature of the body was natural—the circulation equable—the appetite good—the bowels torpid—the urine scanty—respiration inclined to be laborious—head hot to the touch—voice weak. Fluctuation was very evident in the divisions, upon varying the posture of the head. The operation was performed on the 15th October, in the presence of his colleagues. A small, fine, and flat trocar was introduced, in a valvular direction, into the right lateral part of the fontanelle, near the sagittal suture, about an inch and a quarter in depth. The fluid flowed at first, *guttatim*, but afterwards in a gentle stream, without any tinge of blood. Better than

---

* Mr. Money. Med and Phys. Journ. Dec. 1824.

four ounces were drawn off, in the space of five minutes. The child did not seem to suffer—the pulse was at 140. While the water was evacuating the eyes appeared rather more animated. The head was bandaged up after the operation. Nothing particular occurred afterwards. The child appeared to gain flesh, till the 28th, when the head was again tense, and paracentesis capitis was again performed. Three fluid ounces were evacuated this time. To make a long story short, we may state that the head was punctured eleven times within ten weeks, and nearly 40 ounces of fluid removed from the brain. He died on the 7th January 1823, in convulsions.

*Dissection.* The dura mater was much thickened, and adhered very firmly to the inner surface of the cranium. The pia mater was distinctly inflamed. The surface of the brain was smooth, and shewed no appearance of convolutions. On cutting into its substance it was found to be only one sixth of an inch in thickness, yet composed of cortical and medullary matter. There was no vestige of corpus callosum, corpus striatum, thalamus nervi optici, fornix, or septum lucidum. "In short, the various parts usually found in the cerebrum were entirely absorbed, and the space filled up by one large bag, which was lined by an adventitious membrane exceedingly firm, and of about the thickness of ordinary writing paper." This bag was divided into cells by broad flat membranous bands, taking the course of the lobes, and contained about 35 ounces of fluid, "These bands appeared to be plexus choroides, or prolongations of the pia mater." The small apertures made by the trocar could be perceived in the adventitious membrane. The cerebellum was firmer than usual, as also the optic nerves at the fore part of sella turcica. Nothing else worthy of notice.

This case bears, in some degree, on the question, how far the vessels of the brain can be depleted by drawing blood from the general system? It is evident that, although the punctures were made into a cyst containing a large quantity of fluid, only a very small quantity comparatively could be drawn off. The reason is clear. The fluid flowed as long as the cranial envelopes continued to collapse. When the collapse ceased, the fluid refused to issue, on the common principles of hydraulics. This shews that, in the solidified or adult skull, the vessels cannot be depleted in the living body. But then the *pressure* of the blood may be greatly lessened by reducing the whole mass of circulating fluids. It is abundantly evident that no medicine or operation could be of permanent benefit in such a case as the above. Credit is nevertheless due to Mr. Money for having made the attempt, and candidly stated the result.

---

12. *Paracentesis Thoracis.* [Retrospective.] Our readers will remember that in our last No. (3, p. 97) this subject was touched upon, and it was shewn that Laennec was favourable to an operation in cases of watery or other fluids collected in the chest. Sprengel has shewn that the operation is one of the oldest in surgery taking its date in remote mythological periods, and, from the testimony of Cicero and Pliny, owing its first introduction to chance rather than design. Thus, a certain Phalareus or Jason, having been afflicted with an ulcer in the lungs, was deemed incurable by all his physicians. Being desirous of putting an end to his life and afflictions in battle, he received a wound in the side, which wound, instead of proving fatal, opened an impostume, from which a quantity of matter flowed, and recovery was the result. Although this story is rather of the fabulous order, it at least shews that the operation was not unknown to the ancients. That such an operation may often succeed in cases of empyema, there can be little doubt, but hydro-thorax is so much more generally a consequence of other organic disease, than an idiopathic affection itself, that we can have little hope of cure from the mechanical evacuation of the fluid through a wound between the ribs. One of the most remarkable instances of this kind, however, which we have read of, and which is merely alluded to by Sprengel, is related by Moraud in the Memoirs of the Royal Academy of Surgery, for the year 1752. The case was as follows:—

An ecclesiastic, 22 years of age, of good constitution was first affected with a fever and then again with measles. The course of the measles being interrupted, a violent fever ensued, with severe affection of the chest, ending in an œdematous swelling of the left side of the body, difficulty of breathing, and other unequivocal symptoms of hydro-thorax. At this period, Moraud was called in, who gave it as his opinion that there was a collection of water in the left side of the chest, and that nothing but a surgical operation would remove it. This was agreed to, and it was performed by thrusting an ordinary trocar through one of the intercostal spaces, when water rushed out in a full stream, and to the amount of five pints and better. That which came last had a purulent appearance. The patient was instantly relieved; but in the course of a week, the bad symptoms again returned, and in another week it was necessary to have recourse to a similar operation, when five pints more of water were drawn off, mixed towards the end with purulent matter. After this operation, the urinary secretion augmented, the fever diminished, and gentle sweats came out on the surface. The disorder now took a different turn, "as he insensibly fell into so dreadful a marasmus that his life was once more despaired of." A milk and other nourishing diet, however, restored him to perfect health, in which he continued afterwards. A purulent discharge from the wound obtained for several months after the last operation.

Moraud observes that—"the water extracted from the chest being mixed with pus, there was not only a dropsy in this part, but very probably an erysipelas also, at the external surface of the lung or pleura—perhaps at both—which, in rendering the case much more dangerous, must have made the cure more difficult." There can be little doubt but that this was a simple case of inflammatory dropsy of the pleura, and that, if the operation be ever successful, it will be in such cases. The purulent matter, towards the close of each discharge, was no doubt a secretion from the inflamed pleura.

